# An intelligent approach to improve date palm crop yield and water productivity under different irrigation and climate scenarios

**DOI:** 10.1007/s13201-022-01836-8

**Published:** 2022-12-30

**Authors:** Hossein Dehghanisanij, Nader Salamati, Somayeh Emami, Hojjat Emami, Haruyuki Fujimaki

**Affiliations:** 1 Agricultural Research, Education and Extension Organization, Agricultural Engineering Research Institute, Karaj, P.O. Box 31585-845, Alborz Iran; 2 Agricultural Research, Education and Extension Organization, Khuzestan Agricultural and Natural Resources Research and Education Center, Ahvaz, Iran; 3 Department of Water Engineering, University of Tabriz, Tabriz, Iran; 4 Department of Computer Engineering, University of Bonab, Bonab, Iran; 5 Arid Land Research Center, Tottori University, Tottori, Japan

**Keywords:** Water productivity, Yield, Date palm, Subsurface drip irrigation (SDI), ACVO algorithm, ACVO-ANFIS

## Abstract

Drought, rising demand for water, declining water resources, and mismanagement have put society at serious risk. Therefore, it is essential to provide appropriate solutions to increase water productivity (WP). As an element of research, this study presents a hybrid machine learning approach and investigates its potential for estimating date palm crop yield and WP under different levels of subsurface drip irrigation (SDI). The amount of applied water in the SDI system was compared at three levels of 125% (*T*1), 100% (*T*2), and 75% (*T*3) of water requirement. The proposed ACVO-ANFIS approach is composed of an anti-coronavirus optimization algorithm (ACVO) and an adaptive neuro-fuzzy inference system (ANFIS). Since the effect of irrigation factors, climate, and crop characteristics are not equal in estimating the WP and yield, the importance of these factors should be measured in the estimation phase. To fulfill this aim, ACVO-ANFIS employed eight different feature combination models based on irrigation factors, climate, and crop characteristics. The proposed approach was evaluated on a benchmark dataset that contains information about the groves of Behbahan agricultural research station located in southeast Khuzestan, Iran. The results explained that the treatment T3 advanced data palm crop yield by 3.91 and 1.31%, and WP by 35.50 and 20.40 kg/m^3^, corresponding to *T*1 and *T*2 treatments, respectively. The amount of applied water in treatment T3 was 7528.80 m^3^/ha, which suggests a decrease of 5019.20 and 2509.6 m^3^/ha of applied water compared to the T1 and T2 treatments. The modeling results of the ACVO-ANFIS approach using a model with factors of crop variety, irrigation (75% water requirement of SDI system), and effective rainfall achieved RMSE = 0.005, *δ* = 0.603, and AICC = 183.25. The results confirmed that the ACVO-ANFIS outperformed its counterparts in terms of performance criteria.

## Introduction

Date palm scientifically known as *Phoenix dactylifera L.,* is the sixth most important horticultural product in Iran, accounting for about 5.5% of its total horticultural production (Dehghanisanij and Salamati 2017; Agricultural statistics 2018). Due to the specific climatic conditions such as drought, increasing demand for water, decreasing water resources in the southern regions of Iran, implementation of new pressurized irrigation systems and using fertilization in groves seem necessary. The realization of sustainable agriculture in any region requires efficient water management strategies. One of the efficient irrigation systems that have performed positively is the subsurface drip irrigation (SDI) system (Ahmed Mohammed et al. 2020; Mohammed et al. 2021a, b; Alnaim et al. 2022). The main objective of the SDI system is to increase water productivity (WP) (Dehghanisanij and Salamati 2017). Scientific studies show that using the SDI method reduces water consumption by 25–50% for row crops and citrus orchards compared with surface drip irrigation (Davis 1967). In the SDI method, soil moisture during the crop growth period is close to the field capacity (FC) and the crop receives its required water without consuming large energy (Al Wahaibi 2018; Ahmed Mohammed et al. 2020; Mohammed et al. 2021a, b).

The rising demand for agricultural products and difficulties in accessing farm data demonstrate the need to use appropriate models to estimate crop yields and WP. Most input parameters of crop models are not available in Iran. Crop management, crop nutrition, irrigation, soil characteristics, and climatic conditions are among the factors influencing the estimation of yield and energy consumption. Due to the impossibility of simultaneously studying the effects of irrigation, soil, and climate on the crop, efficient WP and yield estimation methods are required (Golabi et al. 2013). Powerful statistical techniques and neural networks have led to the development of yield and WP estimation models (Safari et al. 2019; Bagheri et al. 2012).

Researchers in simulating variables such as the amount of weekly evapotranspiration (Landeras et al. 2009), daily evaporation (Piri et al. 2009), predicting air temperature (Smith et al. 2009), solar radiation (Mubiru 2008), predicting the performance of pressurized irrigation systems (Ababaei and Verdinejad 2013), have used artificial neural networks (ANNs). In recent years, artificial intelligence (AI) methods are powerful alternatives to calculate the yield and WP parameters. Table 1 lists some of the recent studies that employed meta-heuristic algorithms to estimate WP and yield parameters.

**Table 1:** Some key points of intelligent methods for estimating yield and WP

Method	Inference
ANNs (Shirdeli and Tavassoli 2015)	The use of the ANNs can improve the cultivation of saffron in arid and semi-arid regions
Random forest (RF) (Jeong et al. 2016)	The RF algorithm has a high capability in estimating crop yield by considering the minimum number of parameters
An improved genetic algorithm (GA)-back propagation (BP) (Gu et al. 2017)	The GA-BP algorithm describes the relationship between yield and irrigation water under subsurface drip irrigation more accurately
ANNs (Abrougui et al. 2019)	ANNs have good efficiency in estimating crop yield
Boosted tree regression (BRT) and probabilistic neural network (ANN_PNN_) (Zhang et al. 2019)	ANN_PNN_ performs better in modeling the rice yield response function
Radial basis function (RBF) and feed-forward neural (GFF) models (Emami and Choopan 2019)	The RBF model with the input parameter of irrigation water levels could better estimate the barley yield
Fuzzy logic method (Upadhya and Mathew 2020)	This method can be helpful in developing the latest irrigation methods and optimizing yield
Cloud IoT solution (Mohammed et al. 2021a, b)	CSIS validation proved that automatic irrigation of palm trees controlled by sensor-based irrigation scheduling (S-BIS) is more efficient than time-based irrigation scheduling (T-BIS)
Season's optimization algorithm (SO) and support vector regression (SVR) (Dehghanisanij et al. 2021)	The SO–SVR hybrid method has high efficiency in estimating WP and yield
Machine learning algorithms (Rashid et al. 2021)	Machine learning approaches accurately predict Palm Oil yield
A hybrid tree growth optimization algorithm (TGO) and adaptive neuro-fuzzy inference system (ANFIS) (Dehghanisanij et al. 2022)	Based on the TGO-ANFIS model results irrigation with an equal ratio of the well and treated wastewater resulted in improving soil and cotton growth conditions and yield during the study
Supervised learning algorithms (Lad et al. 2022)	Estimating crop stability using monitored algorithms helps to increase farm yield
ANNs combined with sensitivity analysis (Belouz et al. 2022)	The results showed that ANNs provided more accurate predictions of greenhouse tomato yield

Determining the harvest time is one of the main decisions of harvest management. Harvesting sooner or later than optimum date will lead to a reduction in revenue. The purpose of this study is to evaluate the ability of intelligent hybrid approaches based on artificial intelligence in estimating WP and date palm crop yield under SDI for planning at harvest time. It is also possible to select the best possible features from the factors affecting the date palm crop yield using the proposed hybrid approach, and the modeling process using these features.

## Materials and methods

### Case study

**Fig. 1:**
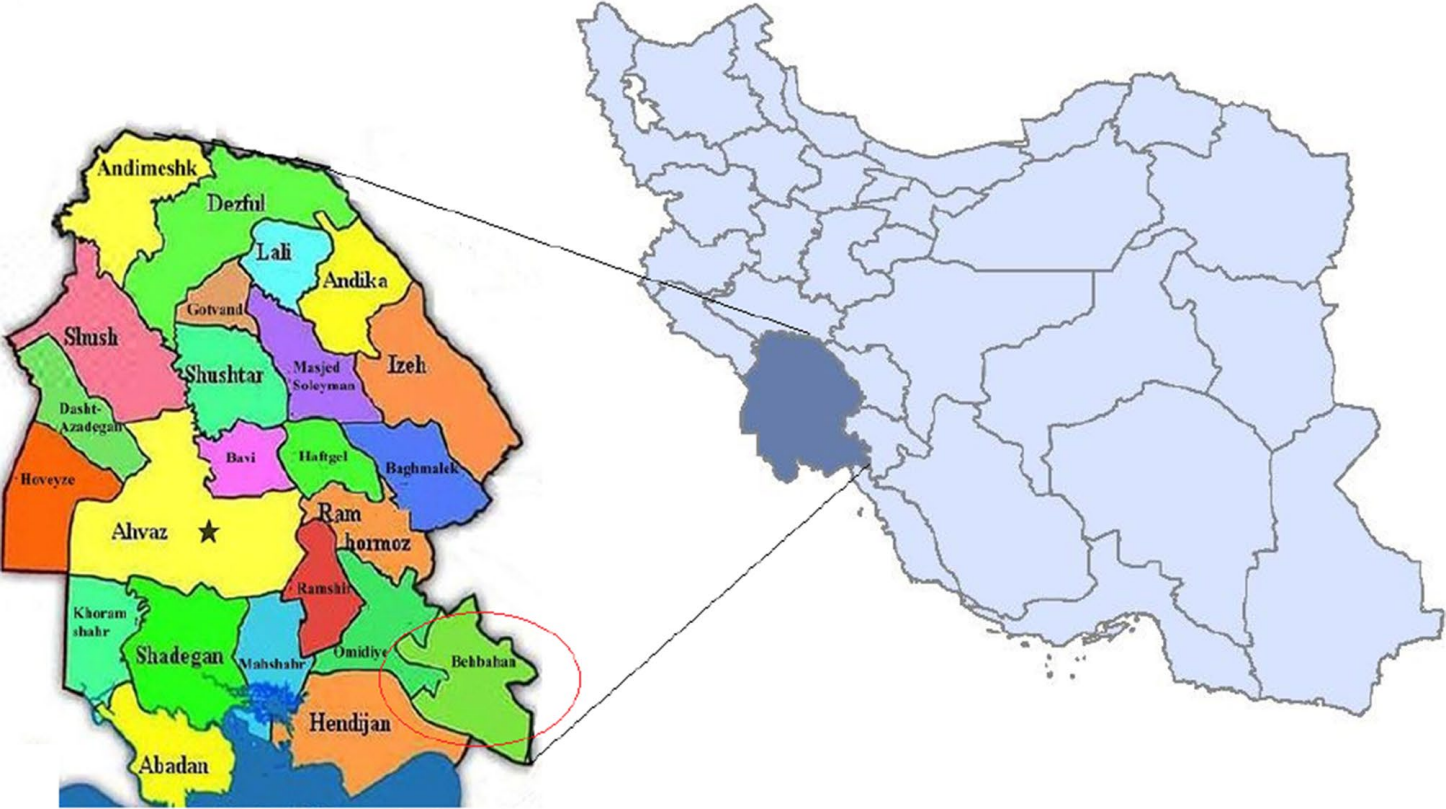
Geographical location of the study area (Ghorbani et al. 2021)

This study was conducted at Behbahan agricultural research station located in Khuzestan, Iran. This station is situated 5 km northeast of Behbahan city at 30° 35'N and 50° 16'E. Its area is 64 hectares; 62 hectares are arable land. Figure 1 shows the geographical location of the study area.

### Methodology

This study was conducted in the form of a randomized complete block design with three replications for 3 years (2013–2016). For irrigation management, SDI system at three levels based on water requirements of 125% (*T*1), 100% (*T*2), and 75% (*T*3) and two palm varieties (Khasi and Zahedi) were considered as main plots and sub-plots, respectively. Date palms were planted as offshoots in 1990. The primary method of irrigating the palms was surface irrigation. In 2013, date palms were equipped with surface and subsurface drip irrigation. The placement of date palms (at planting time) has been implemented in three repetitions. In other words, at the time of planting, the station of date palms was implemented as treatment and replication. Then, SDI treatment was implemented for date palms. Therefore, the date palms are placed in the main plots, and the different irrigation levels treatment placed in the sub-plots. The SDI was 16 mm polyethylene pipes equipped with 4 l/h^−1^ inline pressure compensative emitters 70 cm apart. The subsurface drip pipes were installed 40 cm below the soil surface, one meter from the trunk of the palm tree on each side of the row. The trees received 48 l/h^−1^ through the SDI method since 12 emitters belong to each tree. At the inlet of each SDI line, sensitive flow meters whose resolution was one-tenth of a liter were installed. Installation depth, distance of emitters from each other and tree trunks were determined based on international results and soil texture. The average applied water in *T1, T2, *and* T3* treatments was measured as 1264.80, 1003.88, and 752.88 mm during 3 years, respectively. Zahedi and Khasi varieties are harvested in the form of Khalal and Tamr, respectively. The Zahedi variety is harvested earlier than the Khasi variety (about 10–15 days). Irrigation operation is stopped at the time of harvesting of both varieties. The yield of each tree in each treatment was calculated once all trees had been harvested and weighed. MSTATC statistical software was used to analyze physical characteristics and percentages of fruit moisture and total sugar. The fruit moisture was determined in a vacuum dryer at a temperature of 70 °C according to the AOAC standard method (AOAC 1990). The amount of total sugar and regenerating sugar was determined by Fehling's method (Hosseini 1990). Duncan's multiple range test was used to compare the means of different treatments.

### Irrigation scheduling

The Penman–Monteith equation was used to calculate reference evapotranspiration based on daily data of Behbahan synoptic meteorological station (Allen et al. 1998). Irrigation time was calculated by monitoring the daily meteorological information. Irrigation interval was set at daily. Based on the conducted studies and the FAO 56 model, the crop coefficient was determined (Norouzi and Zolfibavareyani 2010). In Table 2, date crop coefficients during the growing season are presented.

**Table 2:** Crop coefficient for the date

Month	Apr	May	June	July	August	September
Kc	0.91	0.94	0.97	1.00	1.00	1.00

The results of water sample analysis and soil physical and chemical properties are presented in Tables 3 and 4. All measurements and laboratory tests which performed in this study are in accordance with scientific and international standards, such as soil texture determination (ASTM 2007), volumetric soil moisture monitoring (Devices 2008) and water quality analysis (EPA). Table 5 shows the average water consumption of different treatments.

**Table 3:** Quality of the water used for irrigation

Source of water	EC (μs/cm)	pH	Ca^2+^	Mg^2+^	Na^+^	HCO_3_^−^	Cl^−^
Well	3080	7	11.5	9.5	14.5	3	12

**Table 4:** Physical and chemical properties of soil samples

Depth *(cm*)	Mg^2+^	Ca^2+^	Na^+^	HCO_3_^−^	Cl^−^	SO_4_^2−^	pH	Texture	EC (ds/m)	Silt	Sand	Clay
0–30	12.5	31.25	54.34	8.75	5	51.55	8.55	Sicl	5.74	46	7	47
30–60	11.25	36.25	19.02	6.25	6.25	40.98	7.83	Sicl	3.01	42	9	49
60–90	18.75	26.25	40.76	6.25	10	60.68	8.06	Sicl	3.81	48	9	43

EC: Electrical Conductivity; pH: Acidity of water; SO_4_^2−^: Sulfate; Cl^−^: Chloride; HCO_3_^−^: Bicarbonate; Na^+^: Sodium; Ca^2+^: Calcium; Mg^2+^: Magnesium; Sicl: Silty clay loam

**Table 5:** Average applied water in experimental treatments

Month	*P*_e_ (mm)	*T*_1_ (m^3^/ha)	*T*_2_ (m^3^/ha)	*T*_3_ (m^3^/ha)	Total *T*_1_	Total *T*_2_	Total *T*_3_
January	86.60	–	–	–	866.50	866.50	866.50
February	12.00	–	–	–	119.60	119.60	119.60
March	33.00	–	–	–	330.10	330.10	330.10
April	24.80	800.60	640.40	480.30	1048.80	888.70	728.60
May	0.20	1959.90	1567.90	1175.90	1961.60	1569.60	1177.60
June	0.00	2663.40	2130.70	1598.00	2663.40	2130.70	1598.00
July	1.00	2855.10	2284.10	1713.10	2865.10	2294.10	1723.00
August	0.70	2519.00	2015.20	1511.40	2526.30	2022.50	1518.70
September	4.30	1750.10	1400.10	1050.10	1793.20	1443.10	1093.10
October	5.00	–	–	–	49.60	49.60	49.60
November	55.10	–	–	–	551.30	551.30	551.30
December	85.00	–	–	–	850.20	850.20	850.20

*P*_e_: Effective rainfall; *T*_1_: 125% water requirement (in SDI system); *T*_2_: 100% water requirement (in SDI system); *T*_3_: 75% water requirement (in SDI system); Total *T*1, *T*, and *T*3: Applied Water (Irrigation water + *P*_e_).

Water productivity was calculated as follows (Howell 2001):

1 $${\text{WP}} = \frac{{Y\,({\text{usually}}\,{\text{economical}}\,{\text{yield}})}}{{{\text{ET}}}}$$

2 $${\text{ET}} = \,I + \,P + \,D_{P} + \,R_{{{\text{off}}}} \pm \,\Delta S\,$$

3 $${\text{ET}} = \,I \pm \,\Delta S\,$$

where *Y* denotes the economical yield (kg ha^−1^) measured base on the delivered product to the market, ET shows the evapotranspiration (mm), *I* indicated irrigation water measured using a volumetric flow meters (mm), *P* indicates a wetted area (%), *D*_p_ indicates deep percolation (mm), *R*_off_ shows surface runoff (mm), and *ΔS* shows a change in soil moisture (mm).

### Irrigation method

#### Subsurface drip irrigation

**Fig. 2:**
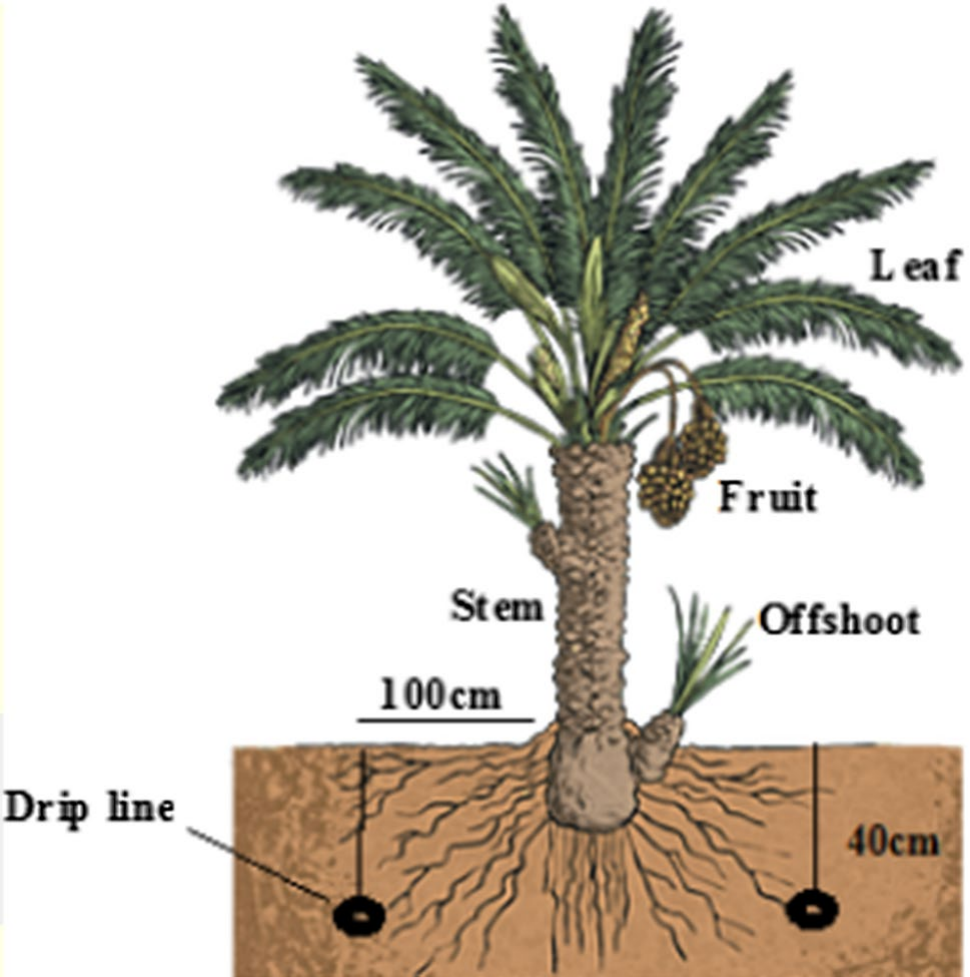
Cross section of the subsurface drip irrigation method

Subsurface drip irrigation system could be a low-pressure, tall proficiency water system framework that employs buried dribble tubes or dribble tape to meet trim water needs. These innovations have been a portion of inundated agribusiness since the 1960s; with the innovation progressing quickly within the final three decades. This is often particularly reasonable for dry, semi-arid, hot, and blustery regions with restricted water supply, particularly on sandy soils (Camp et al. 2000). Figure 2 shows the cross section of the subsurface drip irrigation method (Li et al. 2020).

### Yield and WP estimation methodology based on intelligent methods

#### Anti-coronavirus optimization algorithm (ACVO)

**Fig. 3:**
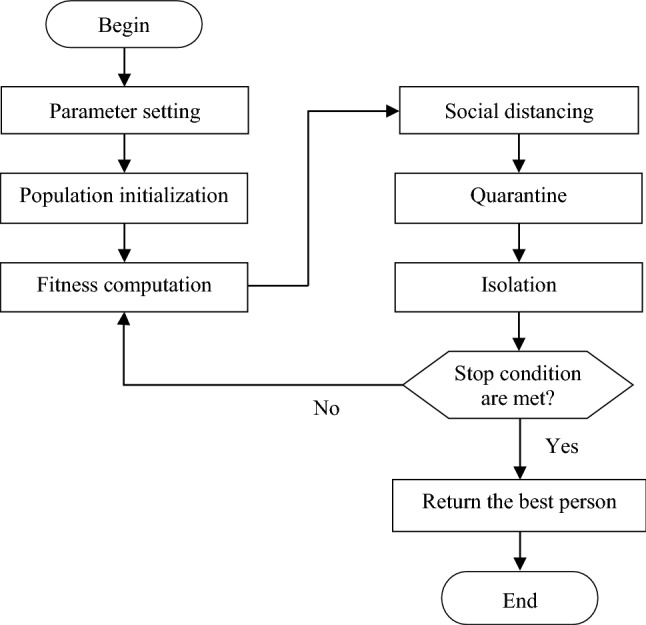
Flowchart of the ACVO algorithm

ACVO is a multi-agent swarm intelligence strategy which is inspired by the containment protocols considered to reduce the spread of the COVID-19 (Emami 2022). Figure 3 shows the flowchart of the ACVO algorithm.

This algorithm is a population-based algorithm which begins its work with a population of solutions. The algorithm is equipped with three operators including social distancing, quarantine, and isolation. The algorithm moves the persons around the solution space and hopefully causes the persons to converge to the global optimum of the cost function. The main principle behind the algorithm is to direct the persons to a safe location in the solution space where the disease transmission is minimal and health protocols are well followed.

In the population creation step, the algorithm generates a collection of solutions. Each solution in the population is referred to as a person. In the social distancing stage, the algorithm attempts to create a safe distance between people in the population.

In the quarantine phase, the suspected individuals with COVID-19 should be monitored to determine whether they are infected or not. In the ACVO, the individuals suspected of having the COVID-19 are those ones that attain low fitness in optimization phase. The suspected individuals should be quarantined for a while to determine the effect of the virus on them. To simulate the quarantine process, the algorithm first selects *q* number of the weakest individuals to form the quarantine list. Then, the algorithm randomly selects some variables from each suspected individual and mutates their values. At the end of the quarantine phase, if the fitness of a suspected individual is equal to or greater than its fitness on the first day of quarantine, then the individual is returned as healthy, otherwise, the individual should be isolated.

In the isolation phase, the algorithm aims to treat infected people so that they can recover their health. The algorithm injects some variables of the fittest healthy individual into the infected individuals. To fulfill this aim, some variables of the best-fit individual are randomly selected and combined with the corresponding variables of the infected individuals. This issue improves the fitness of infected individuals and moves them toward global optimum.

The three phases of social distancing, quarantine, and isolation are applied to the population for predetermined times to improve the fitness of population. Finally, the healthiest individual is considered as the optimal solution to the optimization problem.

#### Adaptive neuro-fuzzy inference system (ANFIS)

The ANFIS, first introduced by Jang (1993), is an efficient kind of multilayer feed-forward ANNs developed based on fuzzy inference system (FIS). ANFIS integrates and makes full use of the advantages of both ANNs and FIS in a unified framework. It is highly adaptive and fast to learn, reflects a nonlinear process structure, and requires less memory. Classical prediction methods are sometimes not able to deal with uncertainty in data (Alarifi et al. 2019). ANFIS is an efficient predictor under such cases. The FIS is build according to the if–then rules, thus the relationship between input and output variables can be identified by regulations and handled uncertainty can be handled easily.

**Fig. 4:**
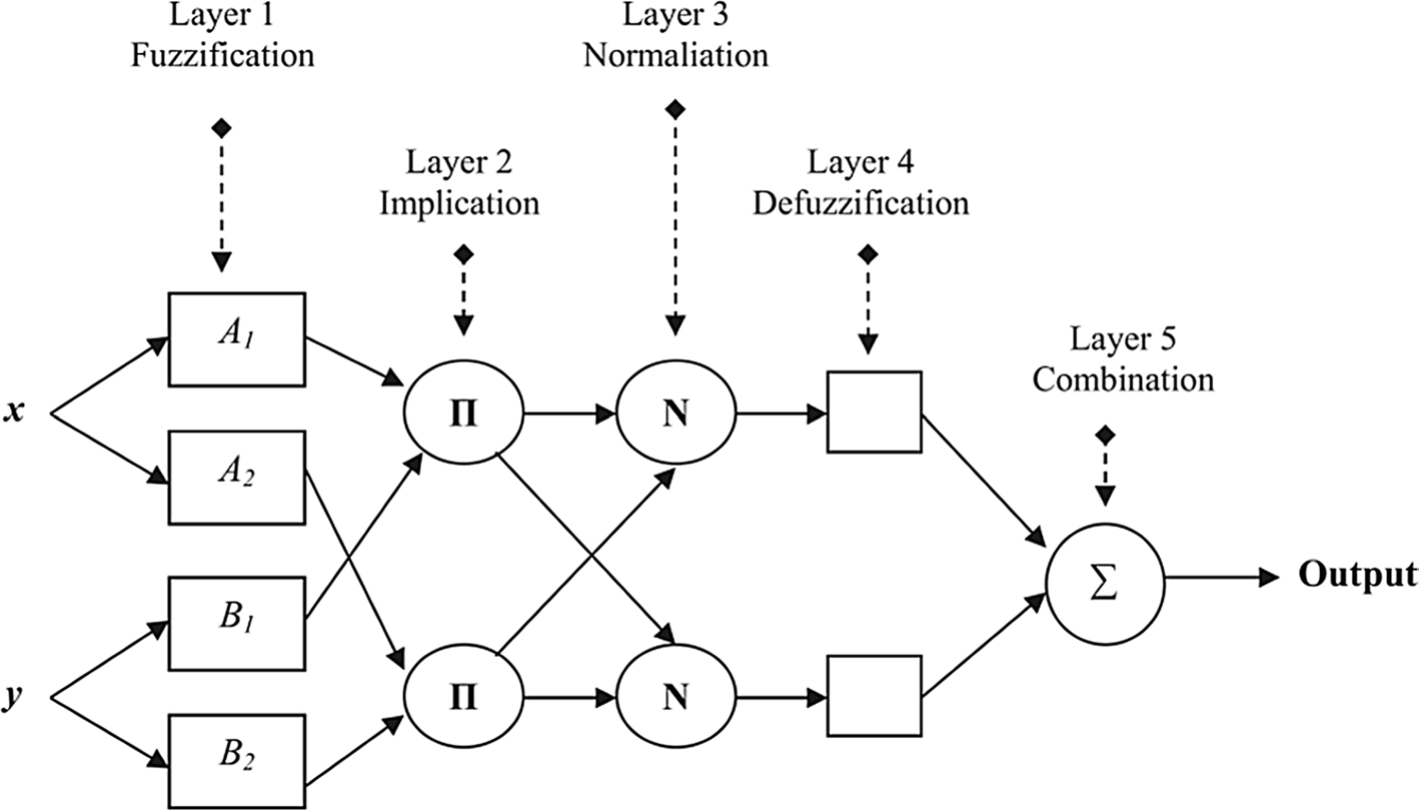
The basic structure of ANFIS

Figure 4 shows the typical architecture of the ANFIS network comprising five layers with two inputs and one output. There five include fuzzification, implication, normalization, defuzzification, and combination. In the ANFIS structure, the nodes are divided into two categories: fixed and adaptable. The nodes of layers 1 and 4 are adaptive, while the nodes of layers 2, 3, and 4 are fixed nodes. The parameters in adaptive nodes can be learnt by optimization algorithms.

To explain the working principle of each layer, we take two fuzzy if–then rules into account as follows:

4 $$R_{1} {\text{: if (}}x{\text{ is }}A_{1} ){\text{ and (}}y{\text{ is }}B_{1} )\,\,\,{\text{then}}\,\,f = p_{1} x + q_{1} y + r_{1}$$

5 $$R_{2} {\text{: if (}}x{\text{ is }}A_{2} ){\text{ and (}}y{\text{ is }}B_{2} )\,\,\,{\text{then}}\,\,f = p_{2} x + q_{2} y + r_{2}$$

where *R* shows each rule, *x*, *y* are the inputs variables, *A*_*i*_ and *B*_*i*_ are fuzzy sets, and *f* is the output of the system. The parameters $p_{i}$, $q_{i}$ and $r_{i}$ are consequent variables that should be determined during the training phase.

In the fuzzification phase, the values of the crisp input variables are modified by membership functions. In this layer, each node generates a membership value of a linguistic label. The node function of the *i*th node may be membership functions such as linear, Gaussian, trapezoidal, triangular or other types. The node function of the *i*th node (*O*_*i*_) using in the Gaussian form can be defined as follows:

6 $$O_{i}^{1} = \mu_{{A_{i} }} (x) = e^{{\frac{{ - (x - c_{i} )^{2} }}{{2\sigma_{i}^{2} }}}} {\text{ for}}\quad i = 1, \, 2$$

7 $$O_{i}^{1} = \mu_{{B_{i} }} (y) = e^{{\frac{{ - (y - c_{i} )^{2} }}{{2\sigma_{i}^{2} }}}} {\text{ for }}\quad i = 3,{ 4}$$

where $c_{i}$ and $\sigma_{i}$ are respectively the center and width of the *i*th fuzzy set *A*_i_ or *B*_i_. These parameters affecting the membership function's shape and should be tuned during the model optimization phase.

The implication phase in layer 2 is responsible to compute the firing weight of rules as follows:

8 $$O_{i}^{2} = W_{i} = \mu_{{A_{i} }} (x).\mu_{{B_{i} }} (y) \, i = 1,{ 2}$$

Layer 3 performs strength normalization for each fuzzy rule as below

9 $$O_{i}^{3} = \overline{W}_{i} = \frac{{w_{i} }}{{\sum\limits_{j = 1}^{2} {w_{j} } }}, \, i = 1,{ 2}$$

The variable *w*_*i*_ is the firing weight of the *i*th fuzzy rule calculated in implication phase.

Layer 4 is devoted to defuzzification phase. Each node at this layer computes a linear function as follows:

10 $$O_{i}^{4} = \overline{W}_{i} f_{i} = \overline{W}_{i} (p_{i} x + q_{i} y + r_{i} )$$

where $\overline{W}_{i}$ is the output of layer 3. The coefficients of $p_{i}$, $q_{i}$ and $r_{i}$ are identified during training phase by minimizing the following equations.

Layer 5 is in charge of combining the output of layer 4 as follows:

11 $$O_{i}^{5} = \sum\limits_{i} {\overline{w}_{i} f_{i} } = \frac{{\sum\limits_{i} {w_{i} f_{i} } }}{{\sum\limits_{i} {w_{i} } }}, \quad i = 1,{ 2}$$

#### ACVO-ANFIS

**Fig. 5:**
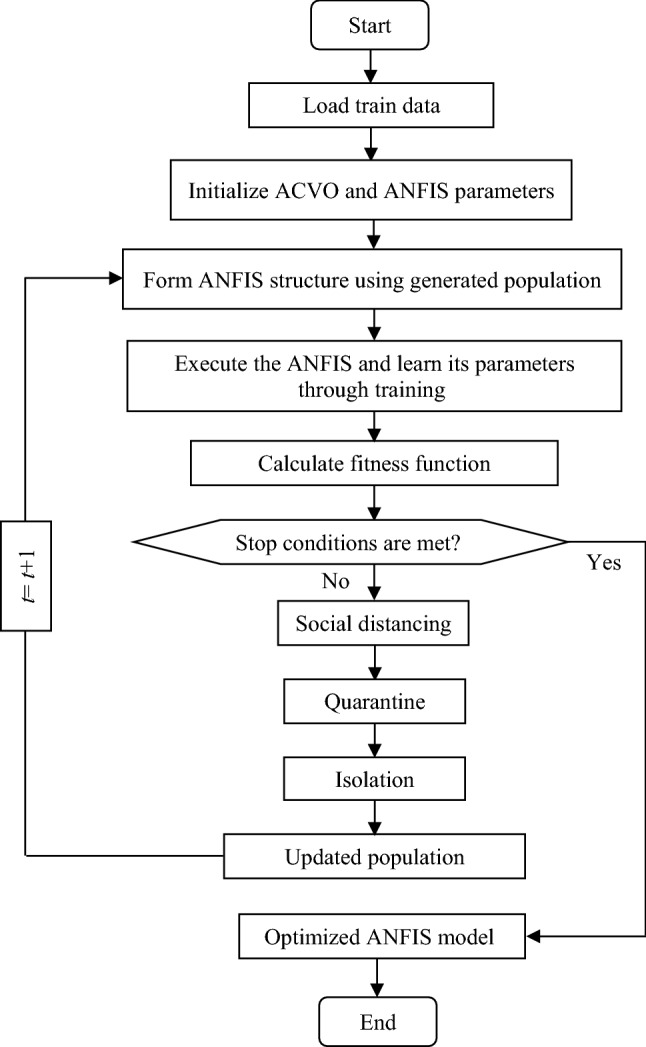
Flowchart of the proposed ACVO-ANFIS approach

Two kinds of structural parameters in ANFIS model are antecedent and consequent parameters that need to be tuned. To optimally tune these parameters, researchers usually used gradient-based methods. The main drawback of gradient-based methods is that they frequently get stuck in local optimality often with slow convergence rate. An efficient alternative is meta-heuristic algorithms that easily can reach to global optimum with high convergence rate. As an element of research, in this paper, we used ACVO algorithm to optimally tune the antecedent and consequent parameters of the ANFIS model. Figure 5 shows the working principle of the proposed ACVO-ANFIS approach.

### Data normalization

To avoid negative effect of different scales of variables on estimation models, it is necessary to correct the data through preprocessing. The data were normalized as follows:

12 $$x = \frac{{x_{i} - x_{\min } }}{{x_{\max } - x_{\min } }}$$

where, *x*_*i*_ is the observed value and *x* is the normal data corresponding to *x*_*i*_. Modeling data were randomly divided into two parts, 80% for the training and 20% for the model test.

## Results and discussion

### Datasets used

**Fig. 6:**
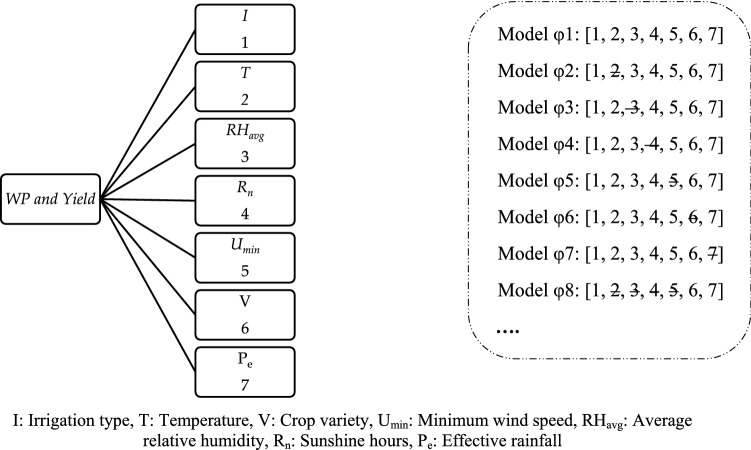
Some input factors in estimating WP and yield

Seven important factors that affect the WP and yield of date palm are irrigation type (*I*), average temperature (*T*), average relative humidity (RH_avg_), sunshine (*R*_*n*_), minimum wind speed (*U*_min_), crop variety (*V*), and effective rainfall (*P*_e_). Since these factors are not of equal importance and may be associated with uncertainty, in intelligent models, the selection of important factors is essential. Table 6 and Fig. 6 present the effective and best-performing factors in estimating WP and yield.

**Table 6:** Effective input factors

Model	Inputs parameters
*φ1*	*I, T, RH*_avg_*, R*_*n*_*, U*_min_*, V, P*_*e*_
*φ2*	*I*, RH_avg_, *R*_n_, *U*_min_, *V*, *P*_e_
*φ3*	*I*, *T*, *R*_n_, *U*_min_, *V*, *P*_e_
*φ4*	*I*, *T*, RH_avg_, *U*_min_, *V*, *P*_e_
*φ5*	*I*, *T*, RH_avg_, *R*_n_, *V*, *P*_e_
*φ6*	*I*, *T*, RH_avg_, *R*_n_, *U*_min_, *P*_e_
*φ7*	*I*, *T*, RH_avg_, *R*_n_, *U*_min_, *V*
*φ8*	*I*, *V*, *P*_e_

### Performance criteria

This section describes the performance criteria, the case study used to evaluate the proposed approach and its counterparts, the comparison algorithms, and the process of feature selection. Four criteria including root-mean-squared error (RMSE), standard deviation (*δ*), and Akaike information criterion (AIC_c_) (Emami et al. 2021) were used to evaluate the performance of the proposed method. Table 7 presents the mathematical formulation of these measures.

**Table 7:** Indicators for evaluation the proposed hybrid approach

Inputs parameters	Model
$${\text{RMSE}} = \sqrt {\frac{1}{n}\sum\limits_{i = 1}^{n} {\mathop {(\mathop j\nolimits_{i} - \mathop i\nolimits_{i} )}\nolimits^{2} } }$$	(13)
$$\delta \% = \frac{{\sum\limits_{{i = {1}}}^{n} {\left| {(i_{i} - j_{i} )} \right|} }}{{\sum\limits_{{i = {1}}}^{n} {j_{i} } }}{ \times 100}$$	(14)
$${\text{AIC}}_{c} = \frac{{2kn + (n\,\ln (\sigma_{\varepsilon }^{2} )(n - k - 1))}}{n - k - 1}$$	(15)

In Eqs. (13–15), $j_{i}$ and $i_{i}$ are the observed and predicted values, respectively. $\overline{j}$ and $\overline{i}$ are average of observed and predicted values. *k* is the number of parameters, *n* is number of samples, and $\sigma_{\varepsilon }$ is the residuals’ standard deviation.

### Quantity features

Table 8 summarizes the combined analysis of variance (ANOVA) of quantitative features of the date palm. The statistical results justify that there was no significant difference between irrigation levels, crop variety, the interaction of irrigation levels and cultivar in fruit weight, fruit flesh to kernel weight ratio, and yield. The results of the ANOVA analysis of WP confirmed that there was a significant difference between irrigation treatments at the level of 5% probability, while there was no significant difference between the two date varieties. The results of mutual analysis of ANOVA of year and crop variety showed that in all quantitative features, there is a significant difference at the level of 1% probability.

**Table 8:** Combined analysis of variance of quantitative features

SV	*df*	Fw (gr)	RFF	NS	NF	Yield (kg)	WP
*Y*	2.00	30.57**	13.81^n.s^	1121.40^n.s^	6,883,570.6^n.s^	54,113,596.60^n.s^	0.380^n.s^
Rep	6.00	0.86**	1.67^n.s^	143.20^n.s^	132,658.70^n.s^	277,650.60^n.s^	0.003^n.s^
*I*	3.00	1.33**	1.35^n.s^	29.60^n.s^	183,132.20^n.s^	4,063,547.40^n.s^	0.193*
Y*I	6.00	5.06**	1.88*	118.10^n.s^	537,312.90**	2,997,609.90**	0.020**
*E*	18.00	1.21	0.65	136.70	125,532.70	459,389.50	0.003
*V*	1.00	32.17**	59.93^n.s^	13,736.50**	11,759,229.30**	6,238,491.50^n.s^	0.043^n.s^
Y*V	2.00	6.34**	17.69**	1001.50**	2,279,572.00**	25,673,504.90**	0.154**
I*V	3.00	0.22**	8.00^n.s^	504.80**	499,934.80^n.s^	1,052,752.40^n.s^	0.008^n.s^
Y*I*V	6.00	0.62**	2.62^n.s^	38.80^n.s^	205,091.00**	3,020,614.20**	0.018^n.s^
*E*	24.00	0.86	1.72	100.20	54,467.00	673,331.00	0.007
*CV*	-	12.28	16.59	14.74	12.65	11.10	14.79

*SV* Sources of variation, *df* Degrees of freedom, *Fw* Fruit weight, *RFF* Ratio of fruit flesh to kernel weight, *NS* Number of strings in a cluster, *NF* Number of fruits in clusters, *Y* Year, *Rep* Repetition, *I* Irrigation, *E* Error, *V* Variety, *CV* Coefficient of variation

**Significant at the level of 1% probability; *Significant at the level of 5% probability; *n.s*. No significant difference

As shown in Table 9, treatment T3 (75% water requirement) with WP = 0.698 kg/m^3^ is superior to treatments T1 and T2. This is likely due to the efficient water utilization of the functional absorbent root zoon (Alnaim et al. 2022). The SSDI system with 75% water requirement is the most appropriate choice for date palm irrigation in arid and semi-arid regions due to its positive effect on WP and yield without changing the chemical quality of the soil (Alnaim et al. 2022). Plant nutrient uptake can be increased and enhanced by appropriate water use within tree systems (Manzoor Alam 1999; Bainbridge 2006; Ahmed Mohammed et al. 2020). The reduction of irrigation water has improved the physical properties of the date palm fruit (Alnaim et al. 2022). Ahmed Mohammed et al. (2020) reported that the SDI system significantly increased data palm crop yield and fruit quality, which is consistent with the results of the present study. Rastegar and Zargari (2011), Alihouri and Tishezan (2011), and Mohebbi and Alihouri (2013), reported that the highest WP was achieved for treatments in which 25% less irrigation was applied. In a similar study, Ahmed Mohammed et al. (2020) concluded that the SDI system has a positive effect on the efficiency of applied water and increasing data palm crop yield in arid and semi-arid regions. Sarhadi and Sharif (2017), showed that the lowest amount of drying damage of date bunch was with the highest applied water, which was consistent with the results of the present study. The length of the fruit has a negative relationship with the amount of applied water. In other words, the reduction of applied water increased the length of the fruit (Sarhadi and Sharif 2017). Alikhani-Koupaei et al. (2018), showed that reducing applied water was effective in increasing fruit sugar content. The number of clusters and fruit moisture had a positive and significant effect at the level of 5% probability on WP. The negative effect of cluster drying on WP was consistent with the results of Sarhadi and Sharif (2017). In Table 8, the values with common letters in a column are not significantly different (*p* < 0.05). The results of this study on WP are consistent with the findings of Mohebbi and Alihouri (2013) and Farzamnia and Ravari (2005). 25% decrease in the water requirement of date palm crop yield did not have any influential changes on WP compared to yield. Mohebbi (2005) and Saleh et al. (2014) showed that applied water of more than 65% of the water requirement caused a decrease in WP, which is consistent with the results of the present study. The superiority of the treatment *T*3 compared to *T*1 and *T*2 treatments can be related to the overestimation of evaporation–transpiration estimation models. Several researchers are trying to provide unknown methods for estimating water requirements or correcting the usual methods, such as Penman–Monteith equation (Schymanski and Or 2017; McColl 2020).

**Table 9:** Comparison of the mean of some quantitative traits and WP at different levels of irrigation

IL	Fw	RFF	NS	NF	Yield	WP (kg/m^3^)
*T*_3_	7.80^a^	8.10^a^	67.80^a^	1757.80^a^	7288.30^a^	0.698^a^
*T*_2_	7.70^a^	7.60^a^	69.70^a^	1990.60^a^	7192.70^a^	0.555^b^
*T*_1_	7.40^a^	8.20^a^	67.20^a^	1824.90^a^	7003.20^a^	0.452^c^

*IL* Irrigation levels of SDI system

Values with a common superscript in each column indicate no significant difference (p<0.05)

### Modeling results

The results of selecting the desired features using the ACVO-ANFIS hybrid approach indicate that the model *φ*_*8*_ with factors of crop variety (*V*), irrigation (75% water requirement of SDI system), and effective rainfall (*P*_e_), with values of RMSE = 0.005, *δ%* = 0.603, and AIC_C_ = 83.25, have the greatest impact on yield and WP. Table 10 presents the results obtained with the ACVO-ANFIS approach. Sensitivity examination appeared that after irrigation, crop variety, and effective rainfall parameters, the average temperature (*T*), minimum wind speed (*U*_min_), and sunshine hours (*R*_n_) parameters are additionally fundamental in estimating the yield and WP. Dehghanisanij et al. (2021), reported that irrigation-fertilizer parameters (PMDI, *F*) and crop variety (*V*) is the most effective parameters in estimating the yield and WP of tomato crops. In a similar study, Sadras and Calvino (2001), showed that irrigation is the most important parameter in estimating soybean and corn yields. Kaul et al. (2005) introduced available water as the most effective parameter in estimating crop yield. Montazer et al. (2010) reported irrigation and rainfall parameters as the most important parameters in estimating wheat yield.

**Table 10:** Evaluation of hybrid approach in WP and yield estimation

Model	Train			Test			Membership functions	
	RMSE	*δ*%	AICc	RMSE	δ%	AICc	c	σ
*φ1*	0.008	0.873	203.20	0.011	1.113	216.90	0.5	0.1
*φ2*	0.010	1.108	206.80	0.014	1.412	239.25	0.4	0.2
*φ3*	0.007	0.860	33.10	0.009	0.880	206.12	0.5	0.1
*φ4*	0.017	1.145	225.50	0.021	1.653	254.60	0.3	0.2
*φ5*	0.014	1.345	216.40	0.017	1.492	241.10	0.7	0.2
*φ6*	0.020	1.598	236.90	0.024	1.650	264.25	0.1	0.1
*φ7*	0.023	1.648	263.90	0.032	1.715	285.30	0.4	0.2
*φ8*	0.004	0.586	16.12	0.005	0.603	183.25	0.5	0.1

**Fig. 7:**
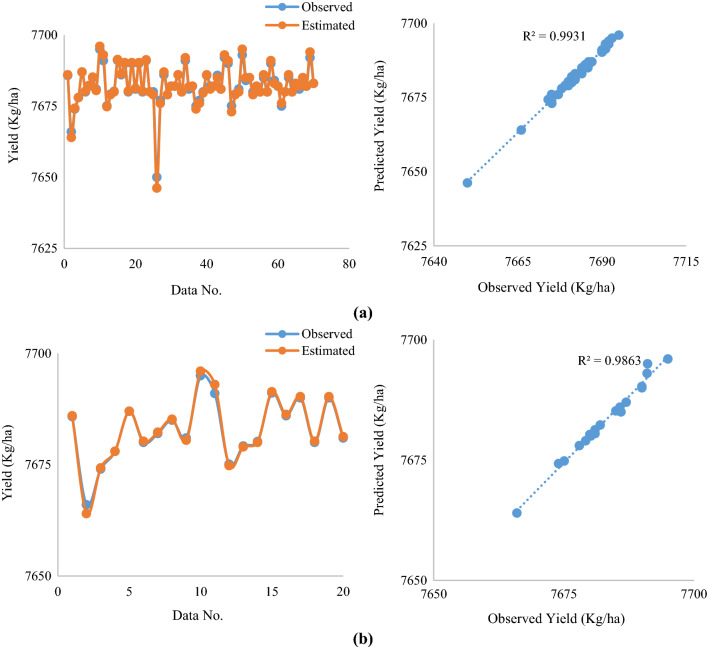
**a**, **b** Comparison of predicted Yield with observed values **a** ACVO-ANFIS on test dataset **b** ACVO-ANFIS on the training dataset

**Fig. 8:**
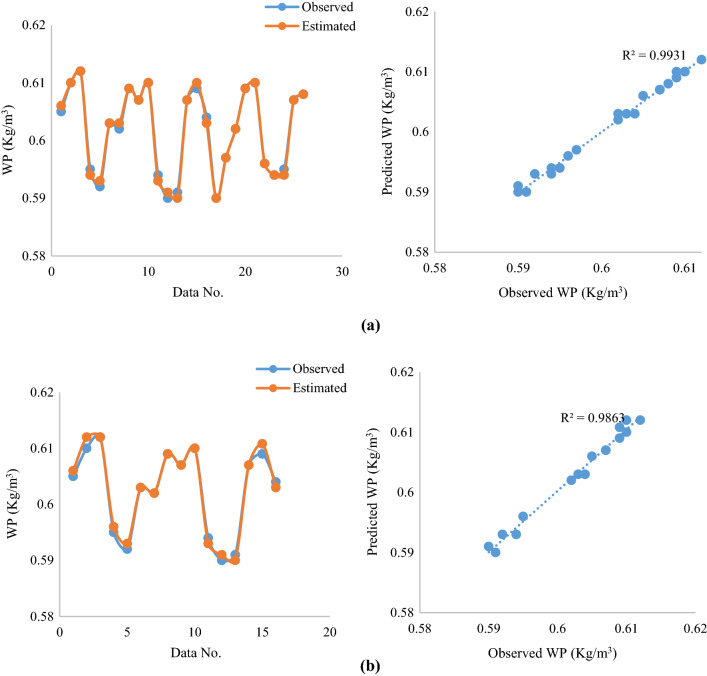
**a**, **b** Comparison of predicted WP with observed values **a** ACVO-ANFIS on the training dataset **b** ACVO-ANFIS on test dataset

Next, modeling of WP and yield estimates was performed by considering irrigation, effective rainfall, and crop variety factors as inputs of the ACVO-ANFIS approach (Figs. 7 and 8).

According to the results, it is clear that the predicted and observed values are in good agreement, which indicates the good performance of the ACVO-ANFIS hybrid approach. Jayashree et al. (2016), predicted sugarcane yield using a fuzzy-neural network (FNN) with a genetic algorithm (GA), imperialist competitive algorithm (ICA), and particle swarm optimization (PSO), the results of which are consistent with the present study.

### Comparison approaches

There are a few approaches in yield and WP estimation using intelligent methods. The proposed ACVO-ANFIS is compared with five state-of-the-art approaches including season's optimization-support vector regression (SO-SVR) (Dehghanisanij et al. 2021), Gaussian process regression algorithm (GPR), (Sharifi 2021), random forest (RF) (Prasad et al. 2021), genetic algorithm-back propagation neural network (GA-BP) (Gu et al. 2017), and ANN (Abrougui et al. 2019). The results rendered by the ACVO-ANFIS approach and other counterparts are compared in Table 11. The results indicate the high efficiency of the ACVO-ANFIS approach with RMSE of 0.005 compared to other similar methods. In general, the ACVO algorithm is a fast convergence algorithm, and surpasses the coequal algorithms in optimizing the ANFIS parameters and thus estimating the data palm crop yield and WP. However, the ACVO-ANFIS approach needs to be parameterized, and the performance of ACVO-ANFIS is scarcely less than perfection. It is suggested that in future analyses, ACVO algorithm be combined with SVR, ANN and other neural network models to increase accuracy and provide generalizable results. Hence, in future analyses, it was offered to combine the ACVO algorithm with SVR, ANN, and other models to improve errors and supply generalizable results.

**Table 11:** Comparison of SO–SVR model with other methods

Model	RMSE	*δ*%
GPR	0.055	–
RF	0.045	–
SO–SVR	0.006	0.614
GA-BP	0.007	–
ANN	0.077	
ACVO-ANFIS	0.005	0.603

## Conclusion

In this study, a hybrid approach based on the ANFIS and ACVO algorithm was proposed to estimate date palm yield and WP under different levels of drip irrigation. The training of the proposed model was performed using data collected from Behbahan agricultural research station. In ACVO-ANFIS, eight models were used to determine the most efficient parameters in estimating and yield and WP. The statistical analysis demonstrated that there is no significant difference between irrigation levels, crop variety, and the interaction of irrigation levels and cultivar in fruit weight, fruit flesh to kernel weight ratio, and yield. The results of selecting the desired features using the ACVO-ANFIS hybrid approach indicate that the model *φ*_*8*_ with factors of crop variety (*V*), irrigation (75% water requirement of SDI system), and effective rainfall (*P*_e_), with values of RMSE = 0.005, *δ*% = 0.603, and AICC = 83.25, have the greatest impact on data palm crop yield and WP. In comparison, the ACVO-ANFIS approach performed better than the practical methods. The results proved that the proposed ACVO-ANFIS approach has promising performance in estimating the yield and WP parameters. The output of the ACVO-ANFIS approach can be developed as a user-friendly mobile application. One of the promising research directions is to test the proposed approach with a large dataset to identify its strengths and weaknesses. Another work is to enhance the operators of the ACVO algorithm to improve the estimation performance of the ACVO-ANFIS approach.

## Data Availability

The data that support the findings of this study are openly available.
